# Advances in immunotherapy for bladder cancer and clinical practice of next-generation sequencing

**DOI:** 10.3389/fimmu.2025.1684597

**Published:** 2026-01-21

**Authors:** Wei Ning, Pengkang Chang, Ji Zheng, Wei Chen

**Affiliations:** 1 Department of Urology, Urologic Surgery Center, Xinqiao Hospital, Third Military Medical University (Army Medical University), Chongqing, China; 2 Department of Urology of Jiangbei Campus, The First Affiliated Hospital of Army Medical University (The 958th Hospital of Chinese People’s Liberation Army), Chongqing, China

**Keywords:** antibody-drug coupling agent, bladder cancer, fibroblast growth factor receptor inhibitors, immune checkpoint inhibitors, next-generation sequencing, trimodality therapy

## Abstract

Bladder cancer kills nearly 170,000 people worldwide each year. Over the past 4 decades, the systematic treatment of metastatic and locally advanced bladder cancer has mainly consisted of platinum-based chemotherapy. In the last 10 years, the development of next-generation sequencing have led to rapid characterization of whole-genome sequencing of bladder cancer, which gives us a better understanding of the pathogenesis of bladder cancer. Based on indications of high mutation burden, and microsatellite instability-high/deficient mismatch repair, immune checkpoint inhibitors have been studied in metastatic and locally advanced bladder cancer as well as bladder-sparing, and shows a good response in these specific indications. Besides, clinically significant expressed molecular targets are used to develop cancer targeted drugs, such as fibroblast growth factor receptor inhibitors and antibody-drug coupling agents. The exploration of molecular characteristics and subtypes of bladder cancer and the development of new drugs and treatment strategy have also stimulated more clinical studies of bladder cancer. Here, we review advances in the treatment of bladder cancer and clinical practice of next-generation sequencing, highlight important advances in immunotherapy for bladder cancer, preliminarily summarize molecular features of bladder cancer for clinical practice, and came up with direction for future treatment development.

## 1 Introduction

### 1.1 Epidemiological investigation

Bladder cancer (BC) is a common malignancy worldwide, with approximately 500,000 new cases and more than 170,000 deaths annually (1). It is one of the three most common tumors of the urinary and reproductive system and is the ninth most common neoplasm and the thirteenth leading cause of cancer death in the world. Men are 3–4 times more likely to develop BC than women (2, 3). The aging of the global population and changes in lifestyle and environment are also affecting incidence and mortality of BC. Increasing risk factors for BC include smoking, infections from schistosomiasis and parasites, occupational exposure to aromatic amines and polycyclic aromatic hydrocarbons, especially with the increasing number of female smokers, the incidence of BC in women has also been increased (4, 5).

### 1.2 Histopathological classification

Based on the depth of tumor invasion, BC is divided into non-muscle invasive bladder cancer (NMIBC) and muscle-invasive bladder cancer (MIBC) (6). Approximately 70% BC patients are NMIBC in clinical, however, high-risk patients with NMIBC have a 50% chance of developing MIBC (7). MIBC has a higher disease-specific mortality (DSM) and a lower 5-year overall survival (OS) (8). BC is also divided into urothelial carcinoma (UC) and non-urothelial carcinoma histologically, more than 90% of cases are UC. Among UC, 10%-55% of patients have varying degrees of histopathological variations and coexist with common urothelial cells in different proportions, including squamous differentiation, adenomatous differentiation, micropapillary, sarcomatoid, nested, plasmacytoid, clear cell-like, lymphoepithelial-like, and adipocytoid. At present the treatment of UC with histopathological variations is the same as that of UC, but efficacy and prognosis is poor, further exploration of treatment strategy for UC with histopathological variations is still needed (9–11).

### 1.3 Traditional treatment model

MIBC accounts for nearly 20% of newly diagnosed cases of BC. Despite promising radical cystectomy (RC) plus pelvic lymph node dissection (PLND), more than half of patients ultimately develop distant metastasis due to disseminated micrometastases. Besides, ileal urinary diversions and ureterocutaneostomy may affect the quality of life (QoL), urinary and sexual function, body image, and mental, social, and emotional health of patients (12, 13). For metastatic MIBC patients, over the past 4 decades, the systematic therapy mainly focused on platinum-based chemotherapy (14). But the benefit from platinum-based chemotherapy is relatively modest, with the 5-6% OS benefit from neoadjuvant chemotherapy after 10 year and no benefit from adjuvant chemotherapy (15). Moreover, approximately 50% of patients with metastatic MIBC are ineligible for platinum-based adjuvant chemotherapy because of impairment of renal function (16). Because of the limited OS benefit and significant renal toxicity, platinum-based chemotherapy is underutilized in the present clinical practice (17). Therefore, there is an urgent need to develop new therapeutic strategies and comprehensive systematic therapies to preserve the bladder and reduce the recurrence rates and improve life quality of this population.

## 2 Trimodality therapy remains a first-line program to preserving the bladder

The exploration of bladder-sparing treatments for patients with MIBC has increased in recent years. With the widespread application of radiotherapy in BC, there is always a lot of interest in bladder-sparing treatments (18). The trimodality therapy (TMT) model is a first-line approach to preserving the bladder recommended by the guide, consisting of maximal transurethral resection of bladder tumor (TURBT), external beam radiotherapy (EBRT), and concurrent chemotherapy (19). It achieves organ preservation and improves survival and QoL (20). However, several retrospective clinical trials have shown no statistically significant differences in OS, disease-specific survival (DSS), and progression-free survival (PFS) between TMT and RC + PLND treatment (21). But patients receiving TMT have higher QoL than those receiving RC + PLND (22, 23). For elderly patients who are unable to tolerate RC because of their poor physical condition, accepting TMT bladder-sparing treatment can get better clinical benefits and QoL (24, 25). The concurrent chemotherapy used in TMT model is usually platinum-based chemotherapy, with carboplatin used in cases of renal insufficiency. Other alternatives, such as 5-fluorouracil and docetaxel, are also available (26–28). However, TMT bladder-sparing treatment also has some issues: patients often develop fibrosis of pelvic tissues after receiving EBRT, which increases the difficulty of salvage RC when cancer recurrence and progression (29). Additionally, EBRT can cause adverse events (AEs) such as radiation cystitis, sexual dysfunction, gastrointestinal symptoms (30). The emergence of image-guided radiation therapy (IGRT) and intensity-modulated radiation therapy (IMRT) can further reduce the AEs of radiotherapy (31).

The TMT bladder-sparing model has been applied in clinical step by step, and there have been many clinical trials that have confirmed higher OS benefit and QoL for patients with MIBC who received TMT compared with RC + PLND treatment, and it also provides data and evidence for new bladder-sparing strategy (32–34). Nowadays in clinical, bladder-sparing treatments, such as TMT and partial cystectomy with neoadjuvant chemotherapy (NAC), have attracted wide interest and extensive attention (35). A systematic meta-analysis assessed DSS and OS after TMT and RC. The mean ten-year DSS was 50.9% for TMT and 57.8% for RC (P = 0.26). The mean ten-year OS for TMT was numerically lower than that for RC (30.9% vs. 35.1%, P = 0.32) (36). This meta-analysis revealed that TMT as a promising treatment option for MIBC may provide comparable survival outcomes to RC. A retrospective study reported that 115 MIBC patients at the Juravinski Cancer Center from 2010 to 2016 experienced TMT or TURBT followed by radiotherapy without concurrent chemotherapy. Complete response (CR) rates of TMT and radiotherapy alone after TURBT were 84.4% and 66.7%, respectively. The three-year disease-free survival (DFS) and OS of patients who received TMT were 68.5% and 49.6%, respectively. The risks of disease recurrence and mortality of patients referred for TMT decreased by 45% and 51%, respectively, compared with those who received radiotherapy alone after TURBT (37). However, TMT for MIBC may result in a significant pelvic recurrence rate (24-43%) and salvage cystectomy rate (25%-30%) (38). Moreover, radiotherapy plus concurrent chemotherapy may damage the bladder wall and lead to gradual fibrosis, which could cause RC finally as well (39). At present, the validation of biomarkers predicting tumor response to radiotherapy plus concurrent chemotherapy and guiding bladder-sparing treatments for MIBC patients was lacking (40) (**Table 1**).

**Table 1:** Clinical trials of TMT for bladder cancer.

Initiator or trial name	Phase	No.	Patient population	Intervention	Key outcomes	Reference
Krasnow RE	retrospective study	303	PUC or VUC	TMT	CR rate was 83% in PUC and 82% in VUC	(11)
Swinton M	retrospective study	287	cN+ M0	RC or TMT	patients received RC or TMT showed no association	(19)
RTOG 9906 (NCT00003930)	phase I/II trial	81	cT2-4a	TURBT+ concurrent chemoradio- therapy+ adjuvant GC	5-and 10-year OS rates were 57% and 36%; 5-and 10-year DSS rates were 71% and 65%	(20)
RTOG 0233 (NCT00055601)	phase II trial	93	cT2-4a	TURBT+ concurrent chemoradio- therapy+ adjuvant CT	5-and 10-year OS rates were 57% and 36%; 5-and 10-year DSS rates were 71% and 65%	(20)
SPARE (ISRCTN61126465)	phase III trial	45	cT2–3 N0 M0	NAC+SBP vs RC	OS was not significantly different between groups	(23)
Giacalone NJ	retrospective study	475	cT2-T4a	TURBT+ concurrent chemoradio- therapy	5-and 10-year DSS rates were 66% and 59%; 5-and 10-year OS rates were 57% and 39%	(26)
Hussain SA	phase I/II trial	41	cT2–4 N0 M0	CT+RT of 55 Gy	CR rate was 25 (71%) at 3-month cystoscopy	(27)
Danesi DT	clinical trial	77	T2-4a N0 M0	TURBT+ HFRT+CT	CR rate was 65 (90.3%); PR rate was 7 (9.7%)	(28)
Kulkarni GS	retrospective study	112	MIBC	RC or TMT	20/56 deaths (35.7%) vs 22/56 deaths (39.3%); 5-year DSS rate was 73.2% vs 76.6%	(32)
Nguyen EK	retrospective study	115	MIBC	TURBT+RT with or without concurrent CT	CR rates were 84.4% vs 66.7%; 3-year OS and DFS rate were 68.5% and 49.6% in TMT	(37)

PUC, pure urothelial carcinoma; VUC, variant urothelial carcinoma; TMT, Trimodality bladder-sparing therapy; CR, complete response; RC, radical cystectomy; OS, overall survival; PFS, progression-free survival; TURBT, transurethral resection of bladder tumor; GC, gemcitabine and cisplatin; CT, chemotherapy; DSS, disease-specific survival; FU, fluorouracil; NAC, neoadjuvant chemotherapy; SBP, selective bladder preservation; RT, radiotherapy; HFRT, hyperfractionated radiotherapy; PR, partial response; MIBC, muscle-invasive bladder cancer; DFS, disease-free survival.

## 3 The emergence of immune checkpoint inhibitors has provided a new choice for patients with bladder cancer

### 3.1 History of immunotherapy

Immunotherapy was first used in UC in the 1970s, and Bacillus Calmette-Guerin (BCG) was used as a common immunotherapeutic agent for NMIBC after TURBT to benefit middle and high-risk patients clinically (41). In UC treatment, immunotherapy has better cancer-control efficacy and lower treatment-related adverse event (TRAE) rates compared to traditional platinum-based chemotherapy (42). Immune checkpoint inhibitors (ICIs), as a new treatment strategy for advanced and/or metastatic UC, provide new choices for patients who are not tolerant to first-line platinum-based chemotherapy, and also provide new treatment options in adjuvant and/or neoadjuvant settings (43). In recent years, the development of ICIs has revolutionized cancer treatments (44). Although immunotherapies affected the immune system and thus caused immune-mediated adverse events (imAEs), these imAEs were generally manageable with early and appropriate interventions (45). Besides, the development of sequencing technologies has improved the genomic characterization of BC, which increased our understanding of the pathogenesis of BC and identified potential therapeutic targets (46). Given the encouraging treatment prospects of ICIs, ICIs have been approved for the treatment of several solid tumors. Especially, accumulating evidence supported the study of ICIs on UC (47, 48). Despite the high mutational burden of BC, a subset of MIBC and advanced BC patients with ICIs still showed durable responses (49). Currently, programmed death receptor 1 (PD-1)/programmed death ligand 1 (PD-L1) and cytotoxic T lymphocyte-associated protein 4 (CTLA-4) are the two checkpoints that has been the most studied in clinical (50).

### 3.2 Exploration of immunotherapy in advanced solid tumors

ICIs has preliminarily revealed anti-cancer efficacy in UC, Hodgkin’s lymphoma, melanoma, non-small-cell lung cancer, gastric cancer, esophageal squamous cell carcinoma, hepatocellular carcinoma, nasopharyngeal carcinoma, and microsatellite instability-high (MSI-H)/deficient mismatch repair (dMMR) solid tumors across clinical studies in China (51). Moreover, it has been approved for the treatment of UC, classical Hodgkin’s lymphoma, non-small-cell lung cancer, and hepatocellular carcinoma in China (52). Additionally, ICIs has positively promising efficacy, acceptable safety profiles and economic benefits, certain new clinical indications in MSI-H/dMMR solid tumors are currently being explored (53). Thus, it may provide a potentially effective weapon against cancer for clinicians and patients, especially for patients with the financial burden. The Food and Drug Administration (FDA) has approved five ICIs (pembrolizumab, nivolumab, atezolizumab, avelumab and durvalumab) for the treatment of MIBC patients who had tumour progression during or following platinum-based chemotherapy (54). Besides, pembrolizumab and nivolumab were also approved as a first-line treatment in MIBC patients who were ineligible for platinum-based chemotherapy and had high expression of PD-L1 (55, 56). PD-1 expresses in activated T lymphocytes, adjusts effector T cell function and suppresses immune responses of T cell (57). As PD-1 is combined with its ligand, PD-L1, T cell activity downregulation and exhaustion occurs. It is now thought that this is one of the mechanisms to prevent autoimmunity (58). In MIBC patients, some studies showed that compared with normal bladder tissue, PD-1/PD-L1 expression was higher in tumor specimens (59, 60). Moreover, PD-L1 expression level in tumor tissue was related to a higher tumor grade and stage, with rich expression in BCG-unresponsive patients (61). Standard treatment for metastatic MIBC has been limited to platinum-based chemotherapy for a long time (62). For some cisplatin-intolerant patients, approval of first-line immunotherapy rely on the PD-L1 expression level, namely Tumor Proportion Score (TPS) and Combined Positive Score (CPS) (63). Employing TPS and CPS Immunohistochemical staining may maximize status for first-line ICIs to treat MIBC patients unfit for platinum-based chemotherapy (64).

### 3.3 The clinical practice of immune checkpoint inhibitors for cisplatin-intolerant patients with locally advanced or metastatic urothelial carcinoma

Currently, platinum-based chemotherapy remains the standard of care for UC (65, 66). Atezolizumab (PD-L1 inhibitor) and pembrolizumab (PD-1 inhibitor) have showed promising efficacy and safety in the IMvigor210 trial and KEYNOTE-052 trial respectively, and recently been approved as first-line treatment for some cisplatin-intolerant patients (67). IMvigor130, a multicentre, phase III, randomised trial, untreated patients with locally advanced urothelial carcinoma (laUC) or metastatic urothelial carcinoma (mUC), were randomly assigned to receive atezolizumab and platinum-based chemotherapy, atezolizumab monotherapy, or placebo and platinum-based chemotherapy. The primary endpoints were PFS and OS (atezolizumab and platinum-based chemotherapy vs placebo and platinum-based chemotherapy) and OS (atezolizumab monotherapy vs placebo and platinum-based chemotherapy). After a median follow-up of 11.8 months, median PFS was 8.2 months in patients receiving atezolizumab and platinum-based chemotherapy and 6.3 months in patients receiving placebo and platinum-based chemotherapy. Median OS was 16.0 months in patients receiving atezolizumab and platinum-based chemotherapy and 13.4 months in patients receiving placebo and platinum-based chemotherapy. Median OS was 15.7 months in patients receiving atezolizumab monotherapy and 13.1 months in patients receiving placebo and platinum-based chemotherapy, there was no statistical significance in OS of two groups of patients. Although OS was not reached, further analysis suggested that patients with high PD-L1 expression might benefit from immunotherapy. These results supported that atezolizumab and platinum-based chemotherapy as first-line treatment prolonged PFS in patients with mUC (68). The KEYNOTE-361 study was used to evaluate the efficacy of pembrolizumab alone or combined with gemcitabine plus cisplatin (GC) regimen for advanced urothelial carcinoma (aUC) patients. For the total population, the pembrolizumab combined GC regimen group did not show a statistically significant benefit in PFS and OS compared to the GC regimen group and for the population of CPS≥10%, the OS benefit of the pembrolizumab group was similar to that of the GC regimen group. Pembrolizumab combined with GC regimen did not show a significant OS benefit in the KEYNOTE-361 study (69). The KEYNOTE-361 study suggested that PD-1 inhibitor and platinum-based chemotherapy did not significantly improve efficacy and should not be widely used for aUC treatment. However, in IMvigor130 study, there was no statistical significance in OS between atezolizumab monotherapy and platinum-based chemotherapy for laUC or mUC. But patients with high expression of PD-L1 could benefit from immunotherapy and PD-L1 inhibitor and platinum-based chemotherapy could prolong PFS in patients with mUC. The KEYNOTE-866 study is a currently ongoing, randomized, double-blind, phase III clinical trial that aims to investigate the efficacy and safety of neoadjuvant therapy (pembrolizumab combined with GC chemotherapy) in MIBC patients, which is expected to provide further evidence and data for the clinical benefit of PD-1 inhibitor combined with chemotherapy (70). The JAVELIN Bladder 100 trial evaluated the efficacy of avelumab (PD-L1 inhibitor) combined with best supportive care (BSC) on maintenance treatment of la/m UC. The results showed that avelumab combined with BSC maintenance had significantly higher median OS and PFS than BSC alone (71) (**Table 2**).

**Table 2:** Clinical trials of ICIs for bladder cancer.

Initiator or trial name	Phase	No.	Patient population	Intervention	Key outcomes	Reference
IMvigor 210 (NCT02108652)	single arm, open-label trial	119	aUC	atezolizumab	BICR-confirmed ORR was 23.5%	(67)
KEYNOTE-052 (NCT02335424)	single arm, open-label trial	370	aUC	pembrolizumab	BICR-confirmed ORR was 28.6%	(67)
IMvigor 130 (NCT02807636)	phase III trial	1213	mUC	atezolizumab+ chemotherapy(A) vs atezolizumab(B) vs chemotherapy(C)	median PFS was 8.2(A) vs 6.3(C) months; median OS was 16.0(A) and 13.4(C) months; median OS was 15.7(B) and 13.1(C) months	(68)
KEYNOTE-361 (NCT02853305)	phase III trial	1010	laUC or mUC	pembrolizumab+ chemotherapy vs pembrolizumab vs chemotherapy	median PFS was 8.3 vs 7.1 months and median OS was 17.0 vs 14.3 months in pembrolizumab+ chemotherapy and chemotherapy	(69)
KEYNOTE-866 (NCT03924856)	phase III trial	870	MIBC	GC+ pembrolizumab or placebo+RC and PLND	pCR rates and EFS	(70)
KEYNOTE-905/EV-303 (NCT03924895)	phase III trial	836	MIBC	pembrolizumab monotherapy or combined EV+ RC and PLND vs RC and PLND alone	pCR rates and EFS	(70)
JAVELIN Bladder 100 (NCT02603432)	phase III trial	147	laUC or mUC	avelumab+BSC vs BSC alone	median OS was 25.3 vs 18.7 months; 1-year OS rates were 78.3% vs. 67.4%	(71)
Ren X	retrospective study	31	laBC or mBC	TGC and GC	median PFS was 36.0 vs 29.0 weeks; median OS was not yet mature vs 48.0 weeks	(74)
ONO-4538-X41 (KCT0003804)	phase II trial	51	cT2-T4a N0	nivolumab+GC+RC	pCR was achieved in 35% (12/34) patients with RC	(75)
Marcq G	phase I trial	8	T2-T4a N0 M0	TURBT+ concurrent RT+ gemcitabine+ atezolizumab	gastrointestinal events were the main toxicity	(76)
MK-3475-992/KEYNOTE-992	phase II trial	6	T2-T4 N0 M0	TURBT+ pembrolizumab+ chemoradio- therapy or chemoradio- therapy alone	1-year bladder-intact DFS rate was 88% in the efficacy cohort	(77)

aUC, advanced urothelial carcinoma; BICR, blinded independent central review; ORR, objective response rate; mUC, metastatic urothelial carcinoma; PFS, progression-free survival; OS, overall survival; laUC, locally advanced burothelial carcinoma; MIBC, muscle-invasive bladder cancer; GC, gemcitabine and cisplatin; RC, radical cystectomy; PLND, pelvic lymph node dissection; pCR, pathologic complete response; EFS, event-free survival; EV, enfortumab vedotin; BSC, best supportive care; laBC, locally advanced bladder cancer; mBC, metastatic bladder cancer; TGC, tislelizumab plus chemotherapy (gemcitabine and cisplatin); TURBT, transurethral resection of bladder tumor; RT, radiotherapy; DFS, disease-free survival.

### 3.4 Immune checkpoint inhibitors bring new possibility for bladder preservation

Postoperative immunotherapy should be considered a potential alternative treatment option for recurrent MIBC patients to achieve bladder preservation (72). Mao J. reported two cases with bladder-sparing treatments for recurrent MIBC. One patient received NAC plus maximal TURBT and postoperative tislelizumab (PD-1 inhibitor). The another was given maximal TURBT combined with postoperative adjuvant intravesical chemotherapy plus tislelizumab (73). In a retrospective study regarding bladder-sparing treatments, 31 patients with locally advanced or metastatic BC received tislelizumab combined with GC or GC alone, respectively. The results suggested that patients treated with tislelizumab plus GC achieved better anti-cancer efficacy and safety than those by GC alone (74). The study (ONO-4538-X41) was a Phase II clinical trial aiming to determine the safety and anti-cancer efficacy of nivolumab (PD-1 inhibitor) in combination with GC as neoadjuvant therapy in patients with MIBC who wanted to preserve the bladder. Preliminary results showed that neoadjuvant therapy of nivolumab combined with GC obtained a better clinical CR rate and longer DFS in most patients (75). There were also clinical trials that combined ICIs with TMT for bladder preservation (76). The MK-3475-992/KEYNOTE-992 trial is an III-phase, multi-center, double-blind, randomized controlled trial conducted at the basis of the TMT model, aiming to explore the safety and efficacy of pembrolizumab combined with radiotherapy and chemotherapy in MIBC patients, with DFS as the primary endpoint. The trial is still ongoing and no primary results have been reported yet (77) (**Table 2**).

### 3.5 Immune checkpoint inhibitors and immune resistance

ICIs have been widely used in neoadjuvant and adjuvant therapy for MIBC at present, but the objective response rate (ORR) is not satisfactory and varies greatly, and immune resistance still needs to be given close attention. The mechanism of immune resistance is complex and not exactly the same as that of traditional chemotherapy and targeted treatments. It involves multiple factors such as the tumor itself, external factors, and the immunity of the body. The currently clearly studied mechanisms include: endogenous tumor mechanisms, exogenous tumor mechanisms and host-related mechanisms. The endogenous mechanisms of tumor mainly involve: gene mutations and abnormalities in signal transduction pathways, including tumor protein P53 (TP53) gene mutation and PTEN/PI3K/AKT/mTOR signal transduction pathway; epigenetic alterations, such as hypermethylation of DNA; other related mechanisms also include the influence of low expression of PD-L1. The exogenous mechanisms of tumor are closely related to the immunosuppressive tumor microenvironment (TME), involving immunosuppressive and effector cells, immunosuppressive factors, alternative immune checkpoints, vascular endothelial growth factors, and TME metabolic reprogramming. Finally, host-related mechanisms are also important influencing factors of immune resistance. Gender, age, genetic history, underlying diseases, and lifestyle of patients are all related to immune resistance in MIBC, while gut microbiota, intestinal metabolism, antibiotics, and hormones can all affect the efficacy of immunotherapy.

## 4 Immune checkpoint inhibitors combined with antibody-drug coupling agent exceeds that of gemcitabine plus cisplatin in the future

Antibody-drug coupling agent (ADC) is a kind of targeted drug based on antigen-antibody specific binding (78). It uses specific linkers to connect antibodies to small cytotoxic drugs, which can effectively act on tumor sites, reduce systemic distribution and increase targeted concentration (79). Once the antibody of ADC binds to the target antigen expressed on cancer cells, ADC will be endocytosed into cells, fused with lysosomes, degraded, and cytotoxic drugs will be released, directly leading to apoptosis (80). In addition, the release of cytotoxic drugs can also alter the tumor microenvironment, further enhancing the efficacy of ADC in killing cancer cells (81). Enfortumab Vedotin (EV) is an ADC drug that targets binding to the tumor-specific highly expressed protein Nectin-4 and was approved by the FDA in 2019 for the treatment of mUC that progresses after platinum-based chemotherapy and/or immunotherapy (82). In clinical trial, EV showed high clinical response. In the treatment of mUC, the efficacy of EV alone or combined with ICIs can completely match or even exceed that of platinum-based chemotherapy (83). In an EV-103 phase Ib/II study, 45 patients with mUC who received 3 cycles of EV combined with pembrolizumab therapy with a median follow-up of 11.5 months showed an ORR of 73.3% and a CR of 15.6%. In 33 patients who had good clinical response, more than half the patients had a sustained response, which suggested that EV combined with pembrolizumab has clinical benefit as an alternative for cisplatin-intolerant patients or as a strategy for patients with mUC who can not tolerate AEs of cisplatin (84). Platinum-based chemotherapy, as a traditional first-line regimen, has AEs such as digestive tract reactions, bone marrow suppression and nephrotoxicity. Some patients cannot tolerate platinum-based regimens due to their own physical conditions or other reasons, especially among the elderly or those with more underlying diseases. The EV-302 clinic trial was designed to evaluate the clinical benefit of EV in combination with pembrolizumab and GC chemotherapy in patients with untreated laUC or mUC. The results showed that in patients with untreated laUC or mUC, treatment with EV and pembrolizumab had significantly better radiographic progression-free survival (rPFS) and OS than GC chemotherapy, with a safety profile consistent with previous reports (85, 86). The emergence of EV has brought a breakthrough in the treatment of patients with laUC or mUC. The combination regimen of EV and pembrolizumab, with its outstanding efficacy and good tolerance, will profoundly change the clinical practice, enabling more patients to benefit from this innovative treatment and bringing new hope for prolonging the OS of patients with laUC or mUC and preserving their bladders for those who require QoL. On December 15, 2023, the FDA fully approved EV and PD-1 inhibitor pembrolizumab for the first-line treatment of patients with laUC or mUC (**Table 3**).

**Table 3:** Clinical trials of ICIs combined with ADC or FGFR inhibitor for bladder cancer.

Initiator or trial name	Phase	No.	Patient population	Intervention	Key outcomes	Reference
EV-101 (ASG-22CE-13-2)	phase I trial	155	Nectin-4 positive mUC	escalating doses of EV	rash, peripheral neuropathy, fatigue, alopecia, and nausea were the most common grade 1–2 TRAEs	(82)
EV-103 (NCT03288545)	phase Ib/II trial	151	laUC or mUC	EV with or without pembrolizumab	the confirmed ORR was 64.5% vs 45.2%	(84)
EV-302 (NCT04223856)	phase III trial	886	laUC or mUC	EV and pembrolizumab vs GC	median PFS was 12.5 vs 6.3 months	(85)
THOR cohort 1 trial (NCT03390504)	phase III trial	266	unresectable UC or mUC with select FGFR3/2 alterations	erdafitinib or chemotherapy (docetaxel or vinflunine)	median OS was 12.1 vs 7.8 months	(93)
THOR cohort 2 trial	phase III trial	351	unresectable aUC or mUC with select FGFRalt	erdafitinib or pembrolizumab	OS was 10.9 vs 11.1 months	(94)
FORT-2 trial (NCT03473756)	phase Ib trial	37	laUC or mUC with FGFR1/3 over- expression	rogaratinib with or without atezolizumab	rogaratinib plus atezolizumab demonstrated a manageable safety profile; RP2D was rogaratinib 600 mg in combination with atezolizumab 1200 mg	(97)

mUC, metastatic urothelial carcinoma; EV, enfortumab vedotin; TRAEs, treatment-related adverse events; laUC, locally advanced burothelial carcinoma; ORR, objective response rate; GC, gemcitabine and cisplatin; PFS, progression-free survival; UC, urothelial carcinoma; FGFR, fibroblast growth factor receptor; OS, overall survival; aUC, advanced urothelial carcinoma; RP2D, recommended phase 2 dose.

## 5 New second-line clinical strategy combined with immune checkpoint inhibitors for locally advanced or metastatic urothelial carcinoma with fibroblast growth factor receptor mRNA overexpression

### 5.1 Advances in fibroblast growth factor receptor pathway

Fibroblast growth factor (FGF)-fibroblast growth factor receptor (FGFR) pathway has a crucial role in embryonic development, angiogenesis and tissue development, and the regulation of homeostasis (87). At the same time, FGF-FGFR pathway also plays an important role in the occurrence and development of malignant tumors (88). FGF-FGFR pathway can cooperate with vascular endothelial growth factor (VEGF) and platelet-derived growth factor (PDGF) to induce angiogenesis in tumor tissues and promote its growth and proliferation (89). An ongoing randomized, open, multicenter Phase Ib clinical trial to evaluate the clinical efficacy of durvalumab combined with FGFR inhibitors in MIBC found no significant differences in OS and PFS between durvalumab combined with FGFR targeted therapy and durvalumab alone (90). Current clinical trial has failed to confirm the efficacy of FGFR inhibitors combined with ICIs, and these findings need to be validated through long-term clinical follow-up and adequate clinical data. Studies have reported the anti-tumor efficacy and mechanism of erdafitinib combined with ICIs in mouse models, as well as compared and evaluated the anti-tumor efficacy between erdafitinib combined with ICIs and erdafitinib alone (91). The results showed that erdafitinib combined with ICIs inhibited tumor growth and had a significant survival advantage in mouse models. The synergistic anti-tumor efficacy of erdafitinib combined with ICIs depended on killing tumor cells directly mediated by erdafitinib, as well as the increase of tumor infiltrating T cells and the decrease of regulatory T cells and the down-regulation of PD-L1 expression on tumor cells through PD-1 inhibitors to enhance anti-tumor activity. Survival rate was also higher in the combined treatment group.

### 5.2 Fibroblast growth factor receptor inhibitor clinical study

Erdafitinib, the most widely studied FGFR inhibitor, is a pan-FGFR1–4 inhibitor and the only FGFR inhibitor approved by FDA for the treatment of aUC or mUC (92, 93). The the randomized, open-label phase III THOR study, which evaluated the safety and efficacy of pembrolizumab in combination with erdafitinib in PD-L1 -positive patients with mUC, found that pembrolizumab in combination with erdafitinib had a significant ORR and prolonged median PFS (94). Final data were not reported, but interim data provided prospect and direction for clinical practice of pembrolizumab in combination with erdafitinib. Like erdafitinib, rogaratinib is also a pan-FGFR1–4 inhibitor. Rogaratinib acts on FGFRs of cancer cells and stromal cells, and its anti-tumor efficacy is mediated by concurrent several mechanisms, such as inhibiting cancer cell proliferation and survival and targeting signaling in the TME (95, 96). The FORT-2 study was designed to evaluate the safety and efficacy of rogaratinib in combination with atezolizumab in first-line treatment for patients with aUC or mUC who were FGFR-positive and cisplatin-intolerant (97). Latest conclusions and relevance showed that in this phase Ib nonrandomized clinical trial, rogaratinib plus atezolizumab demonstrated a good efficacy and manageable safety, suggesting broad potential benefit for patients with aUC or mUC and FGFR mRNA overexpression. Studies of FGFR-targeted therapy combined with ICIs are mostly ongoing, and these findings may provide new clinical strategy for patients with aUC or mUC (**Table 3**).

MIBC exhibits a high frequency of gene mutations, especially FGFR3. Single nucleotide variant (SNV), insertions and deletions (INDEL), amplification and missense mutations of FGFR3 usually lead to overexpression of FGFR3. Approximately 50% of MIBC show overexpression of FGFR3, and FGFR signaling pathway may play a key role in progression of MIBC, thus targeted therapy holds great promise for MIBC. Based on next-generation sequencing (NGS) and targeting the FGFR signaling pathway, targeted therapy may be the most beneficial for MIBC. The FGFR signaling pathway in MIBC has gradually been studied in clinical trials and FGFR inhibitor erdafitinib and rogaratinib have preliminarily shown anti-tumor activity. However, whether targeting the FGFR signaling pathway can improve the prognosis of patients or whether low-invasive MIBC has a better prognosis still needs to be confirmed by further clinical research.

## 6 Next-generation sequencing guides individualized treatment and predicts immunotherapy efficacy and patient prognosis

Recently, the development of NGS has improved the genomic characterization of BC, which may improve the understanding of the genetic underpinnings of disease and drug response for clinicians and allow further customization of treatments and prediction of individualized therapeutic responses (98). Given the unsatisfactory ORR of ICIs as first-line treatment in UC, it is very important to identify biological indicators in predicting the efficacy of ICIs by whole-genome sequencing of BC for further clinical application of ICIs (99).

DNA damage response and repair (DDR) defects play a vital role in the occurrence, development, therapeutic response to immunotherapy, and prognosis of BC (100). Sixty patients with aUC whose tumor samples were analyzed by NGS on pre-immunotherapy tumor specimens met inclusion criteria and thus enrolled in clinical trials regarding immunotherapy. Twenty-eight patients carrying DDR alterations and fifteen harboring deleterious DDR mutations were identified in all patients, respectively. DDR alterations were related to a higher response rate of immunotherapy (67.9% vs. 18.8%, P<0.001) and longer OS and PFS over DDR mutations. A higher response rate was observed in patients whose tumors harbored deleterious DDR mutations compared with patients with DDR alterations (80% vs. 54%, P<0.001) (101).

Ataxia telangiectasia mutated (ATM), a core component of the DDR gene, could recognize DNA double-strand break and activate the ATM-mediated homologous recombination repair pathway (102). It might cause functional alterations of the corresponding encoding proteins and finally affect the sensitivity to immunotherapy (103). A collected cohort data from 210 patients with BC who received immunotherapy showed that ATM-mutant BC patients derived greater OS benefits. It suggested that ATM mutation significantly increased the sensitivity to immunotherapy and had potentially prognostic significance for immunotherapy response (104). More and more DDR-related genes were studied. Teo and his team analyzed 34 DDR genes in several pathways. In patients with mUC receiving nivolumab or atezolizumab, harmful DDR alterations were relevant to longer OS (101). Biallelic mutations of genes of DDR pathways such as TP53 was also significantly related to increasing tumor immunogenicity (105).

The cyclin dependent kinase inhibitor 2A (CDKN2A) gene, also knowed as the P16 gene, encodes multiple tumor suppressor 1 (MTS1) (106). Compared with normal tissue, CDKN2A has a higher expression level in tumor tissue, which can be a biomarker and reflect prognosis in cancers (107). In assays of immune cell infiltration, high CDKN2A expression level in tumor tissue was obviously and positively relateted to more activated immune cells, which suggested that CDKN2A may play a key role in tumor immunity (108). CDKN2A has great potential as a target for immunotherapy. Based on the information and data from the above immunotherapy cohort, we hypothesized that ATM mutation and TP53 mutation as well as CDKN2A mutation could predict immunotherapy efficacy and prognosis in patients with BC.

ARID1B is associated with the metastasis and recurrence of BC as well as immunosuppression (109). It frequently undergoes inactivating mutations in BC and breast cancer. ARID1B is a component protein of the switch/sucrose non-fermentable (SWI/SNF) chromatin-remodeling complex that participates in the occurrence of the tumors by regulating DNA repair and synthesis. Its inactivation may lead to aberrant chromatin closure, preventing the expression of tumor suppressor genes and promoting tumor cell proliferation and evasion of apoptotic mechanisms (110). E2F3 encodes a member of the small family of transcription factors, previous studies have found that E2F3 can promote tumor progression by participating in cell-cycle regulation and activating PI3K/AKT pathway (111). E2F3 is overexpressed in almost all BC, and plays an important role in progression of human BC. Negative regulation of E2F3 in tumor cells can inhibit the proliferation of BC cells (112). Tumor mutational burden (TMB) was correlated with response to ICIs. A retrospective cohort showed that among TMB ≥10 mut/Mb patients, mutations in E2F3 or STK11 correlated with worse OS (113).

Histone methyltransferase KMT2D gene often carries loss-of-function somatic point mutations in BC. It encodes lysine methyltransferase 2D, and deletion of KMT2D is associated with abnormal epigenetic reprogramming in different molecular pathways. KMT2D often mutates in MIBC patients with high histological grade (114). The expression of KMT2D is also regarded as a prognostic biomarker for BC. It promotes the expression of TP53, and TP53 and KMT2D mutation only occurre among patients with MIBC (115). In BC cell lines, overexpression of KMT2D is associated with tumor suppressive effects. It has been observed that patients with high expression of KMT2D tended to have a higher OS and better immune response. However, deletion of KMT2D is associated with cell adhesion, epithelial-mesenchymal transition, and upregulation of various metabolic pathways, including hexose or glycolytic metabolic pathways. Meanwhile, compared with wild-type (WT), deletion of KMT2D have a modest increase in tricarboxylic acid (TCA) cycle and amino acid metabolites (116).

CCND1, namely cyclin D1, is a core gene of the cell cycle and a frequently unregulated biomarker in some tumors. In the early G1 phase, CCND1 acts as a promoter for the progression of the cell cycle from the G1 phase to the S phase or as a growth factor sensor (117). The activity of CCND1 is regulated by Rb protein. CCND1 and/or Rb mutations are frequently observed in BC (118). CCND1 and its partner cyclin-dependent kinases (CDKs) regulate the G1/S transition through Rb phosphorylation. CDK4/CDK6 small peptide inhibitors effectively block Rb phosphorylation *in vivo*. Furthermore, Rb is also phosphorylated by cyclin E-CDK2 in the late G1 phase. The excessive phosphorylation of Rb reduces its affinity for E2F, thereby allowing E2F to activate and transcribe the genes required for cell division (119). When the cell cycle is disrupted, E2F is activated and transcribs genes related to cell proliferation and differentiation, leading to tumor progression. The expression of CCND1 has certain value in terms of diagnosis and prediction of BC, but its clinical significance in the occurrence and treatment of BC have not provided consistent results through relevant research.

## 7 Conclusion and outlook

Currently, the standard first-line neoadjuvant or adjuvant treatment for laUC or mUC remains platinum-based chemotherapy. TMT is a recognized treatment strategy for selected patients with MIBC who are motivated to preserving the bladder. The above data suggest long-term OS benefit for TMT similar to RC, and continue to support TMT as an appropriate alternative for bladder-sparing. The emergence of ICIs has provided a new choice for cisplatin-intolerant patients with laUC or mUC as well as patients with MIBC who want to preserve the bladder and require a higher QoL. The clinical efficacy of ICIs monotherapy or combined with other therapies (such as ADC) on patients with UC has high feasibility and controllable safety. For patients with laUC or mUC who have FGFR mRNA overexpression, FGFR inhibitor erdafitinib may provide new second-line clinical strategy combined with ICIs. At the same time, in the course of treatment, how to choose an more effective treatment program? How to achieve individualized treatment for different patients? How to avoid and manage AEs? Further studies on molecular markers, drug dosage and treatment sequence are needed.

ICIs have been widely used for ten years Has accumulated a great deal of experience in immunotherapy. FGFR inhibitors have a bright future, but they still require a large amount of clinical data support. ADC represented by EV is constantly emerging and have shown positive results in clinical trials. However, to truly achieve precise treatment of UC by ADC, there are still some challenges in terms of safety and drug efficacy. These issues may be addressed through further optimizing and improving linker and coupling technology, developing new hydrophilic small molecule cytotoxic agents, and selecting fully human monoclonal antibody. Meanwhile, the above research results indicate that the clinical benefits of EV have no significant correlation with the level of PD-L1 expression. This suggests that the combination of EV and ICIs can enhance the efficacy and avoid the problem that the effect of ICIs is restricted by PD-L1 expression. Based on the ORR of EV combined with pembrolizumab, EV can play an important role in the preoperative neoadjuvant therapy of MIBC in the future. In addition, identifying effective biomarkers for predicting prognosis and potential UC patients who can benefit from ADC treatment through more precise molecular typing will remain important explorations in the future. We look forward to conducting more extensive research in the future to provide more compelling evidence for clinical treatment of BC.
